# COVID-19 in a Dutch Nursing Home: A Longitudinal Retrospective Care-Home-Level Case Study on Infection Rate, Survival Rate, and Daily Functioning

**DOI:** 10.3390/jcm13010149

**Published:** 2023-12-27

**Authors:** Danielle de Vries, Darwin Röhlinger, Irma Everink, Bjorn Winkens, Joyce Heffels, Adam Gordon, Jos Schols

**Affiliations:** 1 Stichting Land van Horne, Vogelsbleek 1, 6001 BE Weert, The Netherlands; jcf.heffels@landvanhorne.nl; 2 Department of Health Services Research, Care and Public Health Research Institute (CAPHRI), Maastricht University, 6200 MD Maastricht, The Netherlands; i.everink@maastrichtuniversity.nl (I.E.); jos.schols@maastrichtuniversity.nl (J.S.); 3 Department of Methodology and Statistics, Care and Public Health Research Institute (CAPHRI), Maastricht University, 6200 MD Maastricht, The Netherlands; bjorn.winkens@maastrichtuniversity.nl; 4 Academic Unit of Injury, Recovery and Inflammation Science (IRIS), School of Medicine, University of Nottingham, Nottingham NG7 2UH, UK; adam.gordon@nottingham.ac.uk; 5 Department of Family Medicine, Care and Public Health Research Institute (CAPHRI), Maastricht University, 6200 MD Maastricht, The Netherlands

**Keywords:** SARS-COVID-19, nursing home, mortality, infection rate, Barthel Index scores

## Abstract

During the pandemic, nursing homes in the Netherlands were heavily affected by COVID-19. This study assesses the impact of COVID-19 on infection rate, survival rate, and daily functioning over the course of two years among residents of a nursing home in the Netherlands that was amongst the first nursing homes to be affected by the pandemic. This retrospective study followed 70 residents during a two-year period, starting in March 2020. Data were collected on baseline characteristics of participants and the onset, duration, and sequelae of COVID-19 infections. Primary outcomes were mortality and infection rate. The secondary outcome was daily functioning using the Barthel Index at intervals of six months. Within two years, 44 (62.9%) residents were diagnosed with COVID-19. During this study, 72.7% (n = 32) of the COVID-positive residents died, of which 22 deaths were related to the COVID-19 infection. Overall mortality was 60% (n = 42), while COVID-related mortality was 31.4% (n = 22). COVID-19 and multimorbidity (>3 morbidities) were independent risk factors for mortality. Barthel Index scores showed no significant difference in daily functioning. Overall, a high COVID-19 infection rate was seen and was the most common cause of death. COVID-19 did not affect functional status over time.

## 1. Introduction

COVID-19 was first described in December 2019 and reached the Netherlands in February 2020. The first national lockdown began on 18 March 2020. Nursing homes were particularly affected because residents were more vulnerable to infection due to frailty and multiple long-term conditions. Also, cohabitation made the propagation of infection easier. Internationally, Li [1] found that 50% of nursing home residents in Connecticut (US) were infected with SARS-CoV-2 and 40% of all deaths due to COVID-19 in this state occurred in nursing homes during the first wave. Sepulveda et al. [2] studied COVID-19 mortality rates in 12 different countries during this period, Germany, Sweden, Denmark, Netherlands, United Kingdom, France, Spain, United States, Italy, Ireland, Belgium, and Canada, and found that on average 47.3% of all deaths due to COVID-19 occurred amongst nursing home residents.

By April 2020, an average of 60 NH residents with COVID-19 died each day in the Netherlands [3]. Initially, test capacity was low, possibly causing an underestimation of all infections. A second wave of infections started in Autumn 2020 and was associated with even higher mortality rates. Again, older people living with frailty were the most affected [4].

Daily life changed for everyone during lockdowns as people had to stay at home and minimize social contact. Within nursing homes, extra measures were taken. Initially, they closed completely to outside visitors, and social activities within the homes were canceled. Contact between residents was minimized [5].

Several nursing home studies focused on COVID-19 and ways to detect the virus. McMichael [6] studied an outbreak of COVID-19 in a US nursing home, demonstrating a 33.7% mortality rate due to COVID-19. A multinational study by the ECDC Public Health Emergency team [7] studied the epidemiology of the COVID pandemic during the first wave. COVID mortalities amongst nursing home residents had a wide range: 12.7% in Ireland to 82.6% in Belgium. Van Loon et al. [8] found a COVID mortality of 30% in nursing home residents.

Van Loon et al. [8,9,10] found that residents with dementia, chronic kidney disease, and Parkinson’s disease experienced higher mortality following COVID-19. However, the long-term impact of COVID-19 among nursing home residents, its longitudinal influence on functional status, and risk factors for COVID-induced mortality have not been widely studied.

Against this background, we aimed to assess the impact of COVID-19 over a period of two years among 100 residents living in a small nursing home in the south of the Netherlands. This home was severely impacted by COVID-19. The first COVID-19 infection was confirmed on 15 March 2020 and the outbreak spread quickly among residents and staff [11]. In this study, infection rate, mortality rate, and activities of daily living (functional status) among the residents were measured.

## 2. Materials and Methods

### 2.1. Study Design, Setting, and Population

This retrospective longitudinal care-home-level cohort study assessed data from 100 nursing home residents over the period March 2020–March 2022. Those with residency in the home on 1 March 2020 were eligible for inclusion. Legal representatives were approached for consent when a resident had died or could not comprehend the study information due to cognitive impairment.

### 2.2. Data Collection

Data were gathered from electronic care records by first authors DV and DR. Baseline characteristics included age, sex, Body Mass Index (BMI), medical history, multimorbidity (≥3 comorbidities), and whether residents lived on a psychogeriatric (PG) or somatic ward.

The presence of COVID-19 was determined in two ways: either having a positive nasopharyngeal PCR test for the virus; or residents who were clinically highly suspected of being infected and had not been tested due to the low test capacity at the beginning of the pandemic. Residents who had no symptoms and were not tested are categorized as ‘not-tested’. PCR-confirmed re-infections within the study period were also counted.

Data on mortality consisted of the date and cause of death. If a COVID-positive resident recovered from the primary infection but died due to deterioration as a result of sequelae, the cause of death was stated as “COVID-related: secondary”.

Functional status was assessed using the Barthel Index (BI), at baseline and six-month intervals up to 24 months. The BI is a validated observational questionnaire scoring a person’s functioning on 10 different items. A higher score (range 0–20) indicates higher functional independence [12,13]. BI scores were compiled using data from the electronic care plans of individual residents.

Vaccination for COVID-19 started during the study period. Dates and the number of vaccinations were collected.

### 2.3. Statistical Analyses

Numerical and categorical variables were described using means with standard deviations (SDs) and proportions with percentages, respectively. In case of a non-normal distribution, medians and interquartile ranges were used. The independent samples test, chi-square, or Fisher’s exact test were used to compare residents who died during the study period with residents who survived. These tests were also used to compare the COVID-positive group with the COVID-negative group, as well as to the group not tested for COVID-19.

To assess the effect of COVID-19 on mortality, Kaplan–Meier curves were created and compared using the log-rank test. As the testing capacity, and the later occurring vaccinations, differed between the first and subsequent waves, we also analyzed the first wave separately. Multiple logistic regression analysis was used to check whether COVID status (positive PCR or clinically suspect versus COVID-negative versus not tested), age (in years), sex (male versus female), ward (psychogeriatric versus somatic), BI at start of study, and/or multimorbidity (≥3 comorbidities) were independently related to mortality in the first COVID-19 wave (1 March to 1 June 2020).

Linear mixed model analysis was used to analyze the difference in BI between a positive versus negative COVID status at baseline and after 6, 12, 18, and 24 months. A random intercept and random slope at resident level were included to account for the correlation between repeated measures within the same resident. As for the fixed part of the model, time (categorical), COVID status (positive or negative), and their interaction were included.

All analyses were performed using IBM SPSS Statistics for Windows, version 27. Two-sided *p*-values ≤ 0.05 were considered statistically significant.

### 2.4. Data Management and Ethics

The included residents were pseudonymized with the key accessible only to the researchers and held separately to the study database. The pseudonymized database will be saved on a secured drive for 15 years.

The study was approved by the ethical review committee at the Zuyderland Hospital (METCZ20210050).

## 3. Results

### 3.1. Residents

In total, 70 residents agreed to participate (70% response rate). A total of 42 residents (60%) died during the study period (1 March 2020–1 March 2022). These residents were significantly older (87 years versus 81 years), were more often staying in a PG ward (57% versus 29%), had more morbidities (3.5 versus 2.6), more often suffered from multimorbidity (86% versus 43%), and were more frequently diagnosed with dementia (71% versus 40%) (Table 1).

### 3.2. COVID-19

During the study period, 50 residents were tested for COVID-19 using PCR, of which 35 (70%) tested positive. Amongst the 20 residents not tested, 9 were clinically highly suspicious for COVID-19 during the first wave. The remaining 11 residents were not tested and remained without symptoms—they were considered to be negative.

Within the first three months of the study (the first COVID-19 wave), 40 residents were tested or clinically suspected to be positive for the virus. Thereafter, only four additional infections were confirmed, one in January 2021 and three around December 2021.

### 3.3. Mortality

During the first outbreak, 87.5% (n = 21) of all deaths were caused by COVID (Table 2). When looking at the total number of deaths caused by COVID during the total study period (n = 22), it appeared that 95% occurred during the first outbreak. Within the COVID-positive group, 23% (n = 10) died due to a cause that was not COVID-related. These 10 deaths all occurred after the first wave and after these residents had recovered from the infection.

Overall mortality was 60% (n = 42), whereas the COVID-related mortality was 31.4% (n = 22).

**Figure 1:**
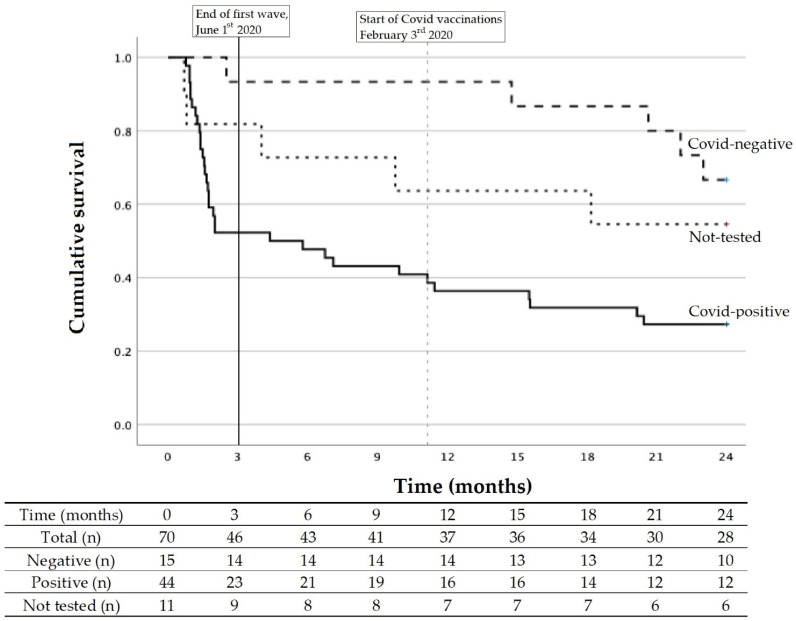
Kaplan–Meier for survival and survival table.

Figure 1 shows the Kaplan–Meier survival curve for the three different COVID groups: PCR-positive, PCR-negative, or not tested. The differences in survival were found to be significant using log-rank analysis (*p* = 0.006).

Deaths in the COVID-positive group totaled 32 (72.7%) for the entire study period. This was significantly higher than in the negative group (n = 5, 33.3%; *p* = 0.006), but not in the not-tested group (n = 5, 45.5%; *p* = 0.148).

Almost half of COVID-positive residents (n = 21, 47.7%) died during the first outbreak. Mortality was lower during this period amongst the COVID-negative (n = 1, 6.7%) and the not-tested residents (n = 2, 22.2%). Regarding the nine clinically suspect residents: all died within a couple of days of becoming severely ill.

During the period after the first outbreak, a total of 30 residents died: 23 (52.2%) within the COVID-positive group, 4 (28.6%) within the test-negative group, and 3 (3.33%) within the not-tested group.

Multiple logistic regression analysis (Table 3) revealed that both being COVID-positive (OR 8.2, 95% CI 1.6–41.9; *p* = 0.012) and suffering from multimorbidity (OR 7.3, 95% CI 1.5–34.4; *p* = 0.012) independently increased mortality risk during the first wave.

#### Vaccinations and Multi-Infections

The surviving residents (n = 39) were offered their first vaccination on 3 February 2021. A total of 34 residents (87.2%) were vaccinated. Five residents remained unvaccinated because they and/or their representatives refused vaccination. The first repeat vaccination took place on 9 December 2021, which was received by 26 (86.7%) of the remaining 30 residents.

During the study period, two residents were infected by COVID-19 twice. They were male, 68 and 92 years old, and became re-infected in December 2021 and February 2022, respectively. Both residents suffered from mild symptoms. Due to the preferences of the relatives, one of these residents had not been vaccinated, the other one had been vaccinated three times by the time of their re-infection.

### 3.4. Level of Functioning/Barthel Index Score

**Figure 2:**
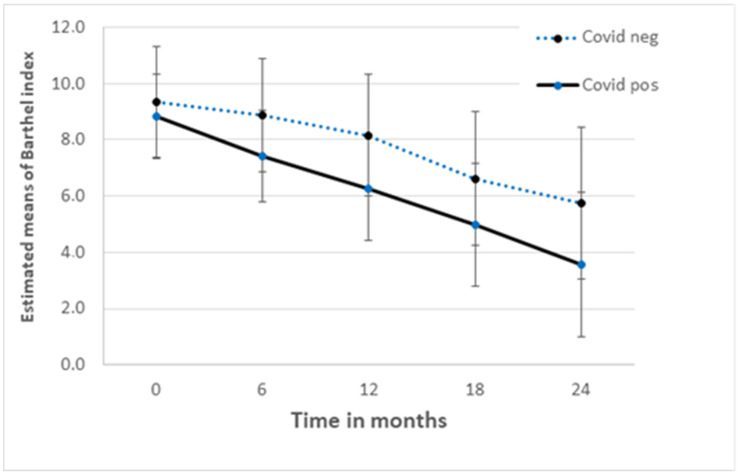
Estimated means obtained from linear mixed model analysis of the Barthel Index with 95% confidence interval.COVID-positive = positive PCR or clinically suspect; COVID-negative = test negative or not tested (and asymptomatic).

Figure 2 shows the course of the BI scores during the study period. BI scores of the COVID-positive group were lower than the scores of the COVID-negative group, although not significantly different at any point in time. Table 4 shows the observed means and estimated mean difference with 95% confidence intervals and *p*-values.

## 4. Discussion

### 4.1. Reflection

The main findings of this study are that residents were severely impacted by COVID-19 with an infection rate of 62.9% (n = 44) and a COVID-19-related mortality rate of 31.4% (n = 22). In the COVID-positive group, 50% died due to COVID-19. COVID-19 was, therefore, the number one cause of death during the study period. In total, 95% of the COVID-19-related deaths occurred during the first wave. During the first wave, COVID-19 and multimorbidity were independent risk factors for mortality. There was no significant difference in Barthel Index scores between residents with and without COVID-19 over time.

The excess of deaths during the first outbreak reflects what was seen in national and international data [2,4,7].

The infection rate within this study could be an underestimation as 11 residents were without symptoms, were not tested, and could have had asymptomatic COVID-19. Van Loon et al. [8,9,10] reported an infection rate of 43.5% (n = 857) among residents of multiple Dutch nursing homes. They found a total mortality rate within the COVID-positive group of 41.9% (n = 534), without attribution of COVID as the primary cause of death. In Spanish nursing homes, Arnedo-Pena et al. [14] found a lower infection rate (36.6%, n = 815) and mortality amongst COVID-positive residents of 21%. Our mortality rate was much higher. This likely represents the fact that they investigated multiple centers and so, incorporated data from homes that were more and less severely affected by outbreaks.

Trevissón-Redondo et al. [15] found that COVID-19 had a significant impact on the Barthel Index. In our study, the difference between the COVID-positive and COVID-negative groups was not significant. Despite the analogous trend we observed, this may reflect that our smaller sample size was less able to detect significant differences between residents who developed COVID-19 versus those who did not. However, it may also reflect that changes made in Dutch care homes affected those with and without COVID in similar ways. These findings have been reflected also in other studies that have suggested functional declines in older people experiencing lockdown, even when they did not contract COVID-19 [16]. Public Health England collected data considering the impact of COVID-19 on older adults, showing an increase in falls and a decrease in functional activity in general [17]. Power et al. [18] found a significant decline in BI amongst COVID-positive nursing home residents in Ireland. During the months following the infection, the BI scores did, however, recover.

A comprehensive comparison of different nursing homes is hard to achieve correctly, particularly across national borders. We can, however, state that the studied nursing home tried to follow the national policies as much as possible. As this nursing home was one of the first to experience the COVID-19 pandemic in the Netherlands, national policies did not yet provide a full guideline on managing the virus. Often available influenza guidelines were used until national COVID-19 guidelines were available, including forming cohorts, confining residents to their rooms, limiting or prohibiting visits, and canceling group activities. It is unknown to what extent this affected the daily functioning of residents, but it could have contributed to a decline. As national policies were updated and refined, lessons were learned about the impact of a lockdown on its population. Therefore a future outbreak would hopefully have less impact than the COVID-19 pandemic has had.

### 4.2. Limits of the Study

This study has some limitations. First, during the first months of the pandemic, the number of infections in the Netherlands surpassed the COVID-19 test capacity [19]. As a consequence, not all residents could be tested in the beginning and the clinically suspected residents were considered and treated as being COVID-19-positive. This could have led to an overestimation of COVID-positivity. However, the nine residents diagnosed on the basis of symptoms showed symptoms compatible with COVID-19 at the height of the pandemic and died within a few days. They behaved very much as if they had COVID-19.

Mortality was significantly higher amongst COVID-positive residents than amongst the confirmed negative residents, resp. 72.7% (n = 32) versus 33.3% (n = 5). This difference might be partially due to different sample sizes (resp. 44 versus 15 residents). It cannot be excluded that some form of ecological bias contributed to the difference. Frailer residents would have been more likely to die and might have been more susceptive to becoming COVID-positive.

The fact that BI scores were not immediately measured but constructed from electronic care records might also be a limitation. However, the method used in our study is comparable to the indirect observation used by Collin et al. [13], and they found this to be equivalent to direct reporting.

The results have to be interpreted with caution due to the small sample size of 70 residents. As this nursing home was the first nursing home that was affected within our organization, early in the national outbreak. By only studying this nursing home, a clear image of the situation within nursing homes at the start of the pandemic was obtained. The study population consisted of 70% of the residents. Informed consent was not given for the remaining 30 patients for unknown reasons. These patients were mostly residents of the somatic ward, thus the percentage of PG residents and the prevalence of dementia is relatively higher in this study. It is generally known that during the pandemic, the impact and incidence of COVID-19 were more pronounced on PG wards than on somatic wards. The PG wards act as a one-household context and maintaining distance is harder.

The willingness of residents and legal representatives to participate in scientific studies is, in general, rather low. As shown by Fincham [20], response rates of sound scientific studies should be expected to be approximately 60% for most research. We acknowledge that the sample size of this study is limited, resulting in less statistical power.

### 4.3. Closing Statement

Over the past year, there has been a significant increase in articles researching COVID-19 among the NH population. With this article, we hope to provide additional insights into the severe impact the pandemic had in this population.

Though lessons were learned from this pandemic, the impact of a new infectious disease will probably still be dramatic. Gaining knowledge of a disease, learning the correct way to protect ourselves, and developing successful treatments and vaccines takes time.

## 5. Conclusions

This nursing home study shows that COVID-19 has had a negative impact on survival. Total mortality was 60% over the course of two years. With a mortality of 31.4%, COVID-19 was the number one cause of death during the two years of the study. Both COVID-19 and multimorbidity were independent risk factors for mortality. No significant differences were found between COVID-positive and COVID-negative residents over time in daily functioning.

## Figures and Tables

**Table 1:** Baseline characteristics of the study population.

	Total (n = 70)	Deceased (n = 42)	Survivors (n = 28)	*p*
Age (years), mean (SD)	84.7 (7.7)	87.2 (5.3)	80.9 (9.2)	<0.001 *
Sex, n (%) Male Female	21 (30.0) 49 (70.0)	11 (26.2) 31 (73.8)	10 (35.7) 18 (64.3)	0.394
Ward, n (%) Psychogeriatric Somatic	32 (45.7) 38 (54.3)	24 (57.1) 18 (42.9)	8 (28.6) 20 (71.4)	0.019
BMI, mean (SD)	25.6 (5.2)	25.3 (5.4)	26.1 (4.8)	0.801 *
Barthel Index, mean (SD)	9.0 (5.0)	8.9 (4.5)	9.2 (5.7)	0.507 *
Number of comorbidities, mean (SD)	3.2 (1.2)	3.5 (1.1)	2.6 (1.2)	0.002 *
Multimorbidity (≥3 comorbidities), n (%)	48 (68.6)	36 (85.7)	12 (42.9)	<0.001
Medical history, n (%)				
Dementia	41 (58.6)	30 (71.4)	11 (39.3)	0.007
Cardiac diseases	58 (82.9)	35 (83.3)	23 (82.1)	1.00 §
Cerebrovascular events	24 (34.3)	11 (36.3)	13 (46.4)	0.081
Epilepsy	7 (10.0)	5 (11.9)	2 (7.1)	0.694 §
Diabetes mellitus	16 (22.9)	11 (26.2)	5 (17.9)	0.416
Pulmonary disease	12 (17.1)	8 (19.0)	4 (14.3)	0.751 §
Morbus Parkinson	9 (12.9)	7 (16.7)	2 (7.1)	0.299 §
Malignancy	15 (21.4)	12 (28.6)	3 (10.7)	0.074
Psychiatric disorder	13 (18.6)	9 (21.4)	4 (14.3)	0.452
Decreased renal function (eGFR < 60)	20 (28.6)	16 (38.1)	4 (14.3)	0.031

Used statistical tests: chi-square. *. Independent *t*-test. §. Fisher’s exact test. *p* < 0.05, statistically significant difference.

**Table 2:** Causes of death during the course of the study, comparing the first outbreak to the rest of the study period.

	Total (n = 42)	During the First Outbreak (n = 24)	After the First Outbreak (n = 18)
COVID-related	22 (52.4)	21 (87.5)	1 (5.6)
Directly	21 (47.6)	21 (87.5)	0
Secondary	1 (2.4)	0	1 (5.6)
Not COVID-related	20 (47.6)	3 (12.5)	17 (94.4)
Cardiac	4 (9.5)	1 (4.2)	3 (16.7)
Cerebrovascular event	2 (4.8)	1 (4.2)	1 (5.6)
Pulmonary	2 (4.8)	0	2 (11.1)
Dementia	3 (7.1)	0	3 (16.7)
Infection (excl. pneumonia)	2 (4.8)	0	2 (11.1)
Fracture	2 (4.8)	0	2 (11.1)
Overall deterioration	3 (7.1)	1 (4.2)	2 (11.1)
Malignancy	2 (4.8)	0	2 (11.1)

**Table 3:** Multiple logistic regression analysis of mortality within the first wave (1 March 2020 to 1 June 2020).

	OR	95% CI	*p*
COVID status (positive versus not-positive *)	8.148	1.584–41.910	0.012
Age	1.076	0.973–1.190	0.152
Sex (male versus female)	1.336	0.344–5.186	0.676
Ward (PG versus somatic)	1.767	0.399–7.824	0.453
Barthel at start of study	1.003	0.875–1.149	0.967
Multimorbidity (≥3 versus < 3 comorbidities)	7.272	1.539–34.361	0.012

* positive PCR or clinically suspect versus COVID-negative or not tested (and no clinical symptoms). OR = Odds Ratio, which was adjusted for the other variables in the model. 95% CI = 95% confidence interval. *p* ≤ 0.05, statistically significant difference.

**Table 4:** Observed means and estimated differences of the Barthel Index at each time point.

Time (Months)	Observed						Estimated		
	COVID-Positive *			COVID-Negative *					
	N	Mean	SD	N	Mean	SD	Mean Difference	*p*	95% CI
0	44	8.8	4.7	26	9.4	5.5	−0.5	0.686	−3.0–2.0
6	22	8.1	4.9	22	8.7	5.5	−1.5	0.266	−4.1–1.1
12	17	7.4	5.5	21	7.9	5.5	−1.9	0.186	−4.7–0.9
18	13	7.5	5.7	20	6.4	5.5	−1.7	0.311	−4.9–1.6
24	11	6.9	5.1	16	5.5	5.7	−2.2	0.243	−5.9–1.5

*. COVID-positive = positive PCR or clinically suspect; COVID-negative = test negative or not tested (and asymptomatic).

## Data Availability

All data used in this study are currently stored by Land van Horne, the Netherlands, and due to internal policy, only available by request, which can be submitted to the researchers and will be evaluated by the internal research committee of Land van Horne, the Netherlands.
